# New Anti-Prelog Stereospecific Whole-Cell Biocatalyst for Asymmetric Reduction of Prochiral Ketones

**DOI:** 10.3390/molecules28031422

**Published:** 2023-02-02

**Authors:** Min-Yu Wang, Shun-Ju Cai, Jia-Chun Lin, Xiao-Jun Ji, Zhi-Gang Zhang

**Affiliations:** 1 School of Pharmaceutical Sciences, Nanjing Tech University, Nanjing 211800, China; 2 College of Biotechnology and Pharmaceutical Engineering, Nanjing Tech University, Nanjing 211800, China

**Keywords:** biocatalysis, whole cells, *Bacillus cereus*, anti-Prelog stereoselectivity, chiral alcohols

## Abstract

The biocatalytic asymmetric reduction of prochiral ketones for the production of enantiopure alcohols is highly desirable due to its inherent advantages over chemical methods. In this study, a new bacterial strain capable of transforming ketones to corresponding alcohols with high activity and excellent enantioselectivity was discovered in a soil sample. The strain was subsequently identified as *Bacillus cereus* TQ-2 based on its physiological characteristics and 16S rDNA sequence analysis. Under optimized reaction conditions, the resting cells of *B. cereus* TQ-2 converted acetophenone to enantioenriched (*R*)-1-phenylethanol with 99% enantiometric excess following anti-Prelog’s rule, which is scarce in biocatalytic ketone reduction. The optimum temperature for the cells was 30 °C, and considerable catalytic activity was observed over a broad pH range from 5.0 to 9.0. The cells showed enhanced catalytic activity in the presence of 15% (*v/v*) glycerol as a co-substrate. The catalytic activity can also be substantially improved by adding Ca^2+^ or K^+^ ions. Moreover, the *B. cereus* TQ-2 cell was highly active in reducing several structurally diverse ketones and aldehydes to form corresponding alcohols with good to excellent conversion. Our study provides a versatile whole-cell biocatalyst that can be used in the asymmetric reduction of ketones for the production of chiral alcohol, thereby expanding the biocatalytic toolbox for potential practical applications.

## 1. Introduction

Enantiomerically pure chiral alcohols are pivotal building blocks for the production of chiral pharmaceuticals, agrochemicals, flavors and other fine chemicals with chiral centers [1,2,3,4]. For example, enantiopure 1-phenylethanol derivatives can be used in the synthesis of ophthalmic preservatives and fragrances [5,6]. Moreover, chiral aromatic alcohols are often used as key intermediates for the synthesis of a series of chiral drugs. (*R*)-3,5-bis (trifluoromethyl)-1-phenylethanol is an important synthon for the synthesis of Aprepitant [7,8]. Ethyl (*R*)-4-cyano-3-hydroxybutyric acid is an important intermediate for the preparation of the cholesterol-lowering drug, Atorvastatin [9]. Currently, chiral alcohols are often prepared through the kinetic resolution of racemic alcohols or the asymmetric reduction of prochiral ketones. Therefore, various chemical catalysts have been developed in recent decades for this purpose [10,11,12,13]. Compared with conventional chemical catalysis, biocatalysis is attracting increased attention due to its inherent advantages, such as its remarkable enantioselectivity, relatively mild reaction conditions and environmental friendliness [1,14,15].

**Scheme 1:**
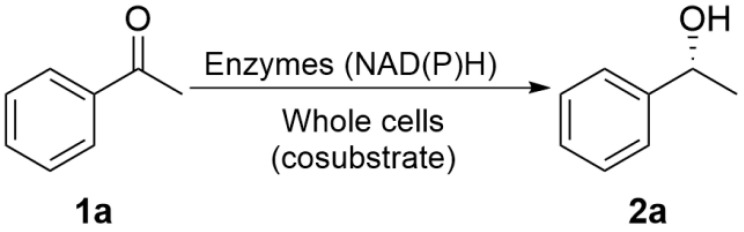
Biorcatalytic anti-Prelog reduction of acetophenone for (*R*)-1-phenylethanol.

Biocatalytic asymmetric reduction of prochiral ketones for chiral alcohols can be performed by using isolated enzymes and whole cells. Compared with enzymatic processes, microbial whole cell catalyzed reactions are preferable and are often well accepted for practical reasons [16,17,18]. Using whole cells as catalysts avoids the need of enzyme purification and is more convenient. In addition, cells not only have endogenous cofactor regeneration systems but can also provide a more stable natural environment for reductase [19,20]. Thus, many efforts have been undertaken regarding the synthesis of chiral alcohol via the asymmetric reduction of prochiral ketones using wild-type microbial cells or recombinant cells as biocatalysts [21,22,23,24]. Moreover, the number of various new whole cell biocatalysts with excellent activity and stereoselectivity for the asymmetric reduction of ketones has steadily increased in recent decades; for example, a newly isolated strain of *Rhodotorula* sp. AS2.2241 was used as a whole-cell biocatalyst in the synthesis of enantiopure (*S*)-1-(4-methoxyphenyl) ethanol from asymmetric reduction of 4′-methoxyacetophenone with 99% *ee* [25]. The immobilized *Rhodotorula* sp. AS2.2241 cells showed improved stability and solvent resistance in biocatalysis [26]. Recently, the catalytic capacity of a panel of marine-derived fungi was evaluated with the purpose of discovering new whole-cell biocatalysts. Good activities and excellent enantioselectivities with 99% *ee* towards structurally related aromatic ketones were achieved by some of the examined marine fungi [27]. Although substantial progress has been achieved, most of the known biocatalysts, whole cells or isolated enzymes used in the asymmetric reduction of prochiral ketones to chiral alcohols generally follow Prelog’s rule [28,29]. Ketone reductases or cells with anti-Prelog stereospecificity are still very limited in number, and anti-Prelog chiral alcohols are in great demand in organic synthesis and the pharmaceutical industry [30]. However, only a few reductases or whole-cell biocatalysts with anti-Prelog stereopreference have been reported in the literature [31]. Thus, the discovery of new biocatalysts with anti-Prelog stereopreference, either whole cells or isolated enzymes, is crucial (Scheme 1).

In this study, we report a new isolated bacterial strain of *Bacillus cereus* TQ-2 with good enantioselectivity for the biocatalytic reduction of prochiral ketones for the production of chiral alcohols. The resting cells of TQ-2 exhibit high activity and excellent anti-Prelog enantioselectivity toward the model substrate, acetophenone. The catalytic properties of TQ-2 whole cells and the key factors of the reaction conditions were investigated in detail. In addition, the cells were highly active to a panel of structurally diverse ketones and aldehydes, indicating a broad substrate spectrum and good catalytic activity as a new whole-cell biocatalyst.

## 2. Results and Discussion

### 2.1. Screening of Strains with Ketone Reduction Activity

**Figure 1:**
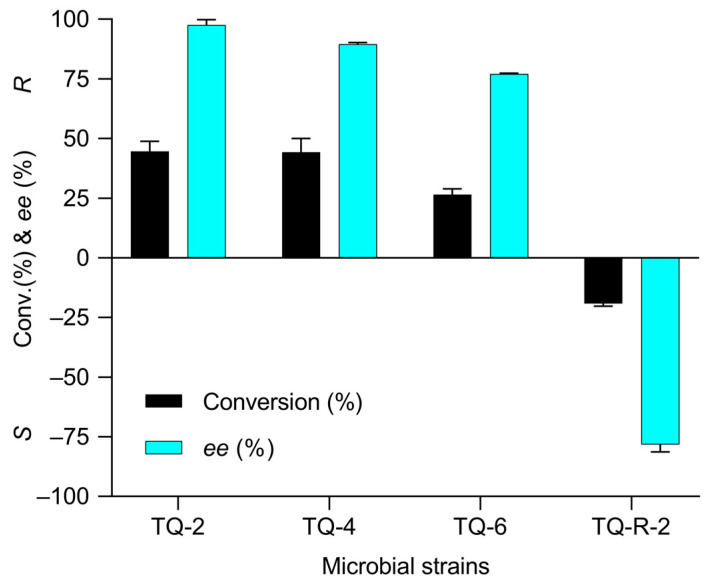
Microbial strains isolated from soil samples for selective reduction of acetophenone. Reaction conditions: 2 mM of acetophenone, 0.1 g/mL of wet cells, 24 h, 600 rpm. The alcohol products were analyzed by GC.

**Figure 2:**
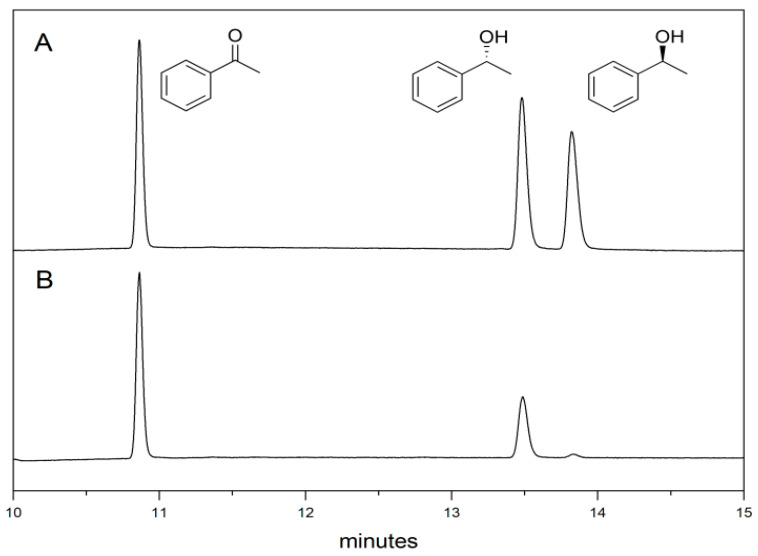
Microbial asymmetric reduction of acetophenone using whole cells of TQ-2 strain. (**A**): the standard substrate and alcohol products; (**B**): the products of acetophenone conversion catalyzed using TQ-2 whole cells.

With the aim of screening for microorganisms with ketone reduction activity, strains with considerable transformation activity to the model substrate acetophenone were obtained after several rounds of enrichment processes. Based on the catalytic activity, four strains exhibiting high catalytic activity were further evaluated for their ability to reduce acetophenone into alcohol, leading to conversions with a range from 19% to 45%. In addition, we were delighted to discover that strains TQ-2, TQ-4 and TQ-6 exhibited anti-Prelog stereoselectivity, achieving *ee* values ranging from 78% to 99% (Figure 1). Among them, the whole cells of the TQ-2 strain converted acetophenone into (*R*)-1-phenylethanol as the sole product, exhibiting the best conversion and excellent enantioselectivity (Figure 2). Thus, the TQ-2 strain was subsequently applied to microorganism identification based on its physiological characteristics and 16S rDNA sequence analysis. Meanwhile, the catalytic properties of its resting cells were also investigated in detail.

### 2.2. Identification of TQ-2 Strain

**Figure 3:**
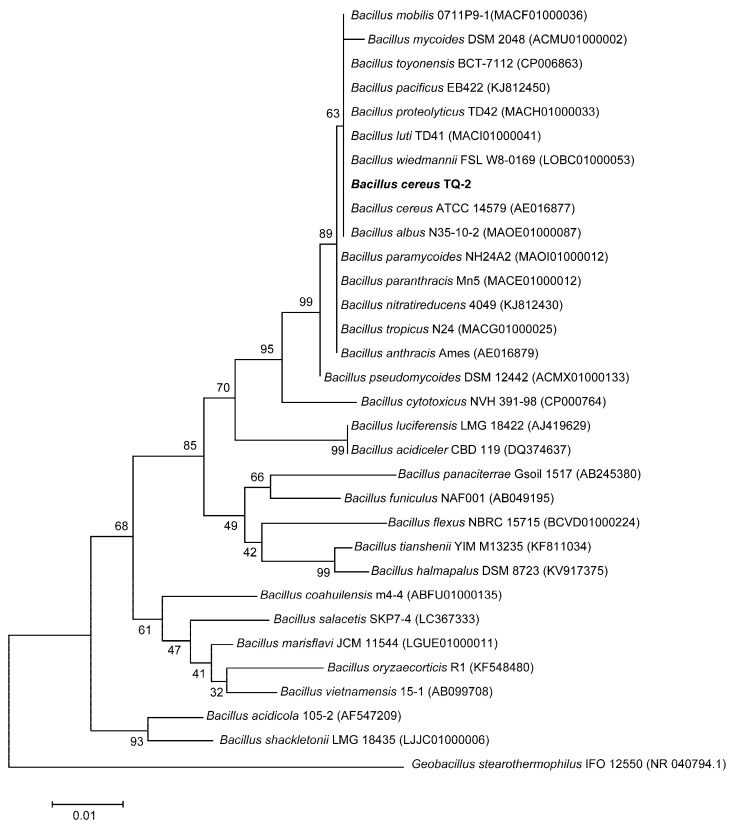
The phylogenetic tree based on 16S rDNA sequences, exhibiting the revolutionary status of *B. cereus* TQ-2 among related taxa. The tree was constructed by the neighbor-joining method, and the numbers in parentheses represent the sequences accession number in the GenBank database.

The isolated TQ-2 strain is a facultative anaerobic, Gram-positive and rod-shaped bacterium. The strain can form spores under certain growth conditions. The results of the physiological and biochemical tests conducted for strain identification are summarized in Table 1. The TQ-2 strain was able to produce arginine dihydrolase and gelatinase, and it makes use of ribose, glucose, fructose, maltose and trehalose as carbon sources for growth. The strain exhibited positive esculin hydrolysis. In addition, the 16S rDNA sequence of the TQ-2 strain (1430 bp, GenBank accession no.OP035935) was determined (Figure S1 in Supplementary Materials), and a phylogenetic tree was constructed (Figure 3). It was found that strain TQ-2 was closely clustered with *Bacillus cereus* ATCC 14579 (AE016877), having sequence identities of 99% (Table S1). Thus, the TQ-2 strain was assigned to the genus Bacillus. Although 16S rDNA gene sequencing has long been considered the gold standard for bacterial identification, it sometimes faces challenges in distinguishing closely related strains at the species level. In recent years, some genetic markers with much higher resolutions have been frequently employed in the identification of closely related strains at the species level, producing produced numerous reference sequences in public databases [32].

It is considered that the *gyrB* gene could be used as a highly effective phylogenetic marker for the identification of closely related species compared to the 16S rDNA sequence [33]. In this study, the *gyrB* gene sequence of TQ-2 was determined (Figure S2, Table S2), and was used for the identification and phylogenetic analysis of species of the *Bacillus* group. In addition, the results from the database searches in relation to the MALDI-TOF mass spectrum of the strain proteins indicated that TQ-2 was a *Bacillus cereus* strain (Figure S3). Thus, based on the results of the phylogenetic analysis and phenotypic tests mentioned above, the strain belongs to *Bacillus cereus* and was designated as *Bacillus cereus* TQ-2.

Currently, a number of anti-Prelog specific whole cell biocatalysts have been discovered in different microbial species, including *Empedobacter brevis* ZJUY-1401 [34], *Acetobacter pasteurianus* GIM1.158 [35], *Acetobacter* sp. CCTCC M209061 [36], *Torulaspora etchellsii* 20126 [37], *Trichoderma asperellum* ZJPH0810 [38], *Lactobacillus kefir* [39], *Geotrichum* sp. [40], *Yarrowia lipolytica* [41], *Lactobacillus brevis* [42] and *Oenococcus oeni* [43]. Several enzymes with excellent anti-Prelog stereoselectivity that can be used in biocatalysis processes have been isolated [7,31]. The Bacillus species are widely distributed in the environment and has high biotechnological potential in bioremediation and biodegradation, the production of useful enzymes and biotransformation. Recently, whole cells and isolated enzymes of *B. cereus* have been successfully employed as biocatalysts in biotransformation processes for the production of valuable chemicals. Tang et al. explored a newly isolated *B. cereus* zju 4-2 strain for use in the efficient transformation of vitamin D3 to 25-hydroxyvitamin D3, providing a high yield [44]. Zhang et al. reported a new biocatalytic route via *Bacillus cereus* WZZ006 whole cells for the preparation of (*S*)-5-chloro-1-oxo-2,3-dihydro-2-hydroxy-1H-indene-2-carboxylic acid methyl ester, an important chiral intermediate of indoxacarb synthesis [45]. However, to the best of our knowledge, this is the first time that *Bacillus cereus* was reported as whole cell biocatalyst for use in the production of anti-Prelog chiral alcohols from prochiral ketones. The discovery of *B. cereus* TQ-2 with anti-Prelog stereopreference is of significance not only for meeting the demands of chiral alcohol synthesis, but also for exploring the distribution of microorganisms that can be used in the bioreduction of ketones following anti-Prelog’s rule in nature.

### 2.3. The Effect of Reaction Temperature and Reaction Time on Microbial Selective Reduction by B. cereus TQ-2

**Figure 4:**
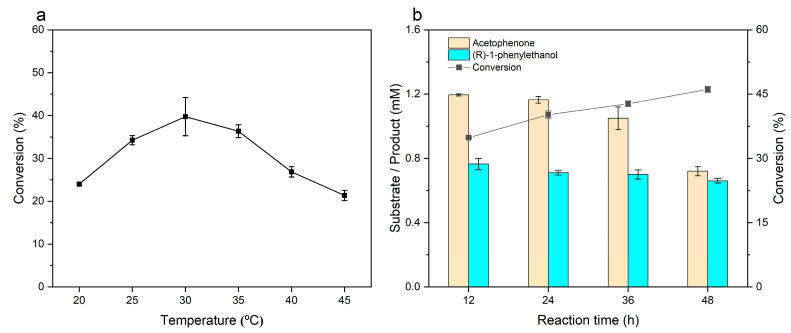
Effect of temperature (**a**) and reaction time (**b**) on the *B. cereus* TQ-2-catalyzed bioreduction of acetophenone. The general reaction conditions used otherwise stated: 2 mM of substrate, 0.1 g/mL of microbial cells, 5 mL of phosphate buffer (100 mM, pH 7.4), and 600 rpm; (**a**) increased reaction temperature from 20 °C to 45 °C; (**b**) prolonged reaction time from 12 h to 48 h.

The reaction temperature and reaction time are critical parameters in biocatalytic reactions due to the biological properties of isolated enzymes or whole cells. Thus, the effect of the reaction temperature on the catalytic activity of TQ-2 cells was examined. As shown in Figure 4a, the *B. cereus* TQ-2 cells showed considerable activity at a broad temperature range from 20 °C to 45 °C. The best acetophenone conversion of 39.8% was achieved at 30 °C. Meanwhile, a conversion of 21.8% was obtained at 45 °C. In addition, by increasing the reaction time to 48 h, the conversion was improved slightly, reaching 49.1%. Nevertheless, the concentration of (*R*)-1-phenylethanol product in the reaction system decreased slightly rather than increased over reaction time (Figure 4b). The prolongation of the reaction time from 12 h to 48 h resulted in the concentration of the remaining substrate decreasing from 1.1 mM to 0.7 mM compared with the control experiments (Figure S4). After scrutinizing the conversion results, we found that the increased conversion was due to the loss of acetophenone caused by possible microbial consumption. Considering the complex metabolic pathways of microorganisms, the probable consumption of a substrate or product by whole cells is not surprising, especially as cells from a wild-type strain were used as biocatalysts in the biotransformation process [46,47,48]. Thus, it is necessary that the related enzymes in the cells of *B. cereus* TQ-2 need to be identified, isolated and characterized for the efficient and selective reduction of ketones. 

### 2.4. The Cofactor Preference and Optimum Reaction pH of TQ-2 Whole Cells

**Figure 5:**
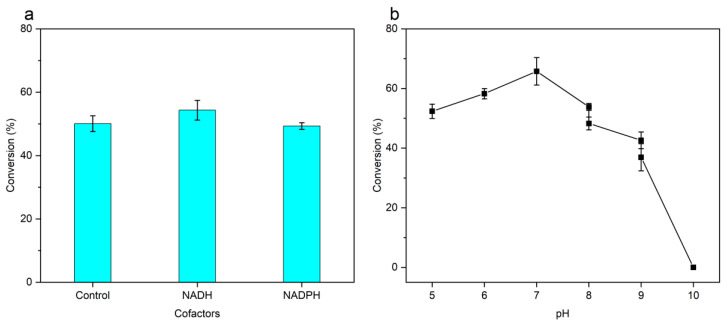
The cofactor preference of TQ-2 whole-cell biocatalyst (**a**) and the effect of pH on the reduction of acetophenone (**b**). The general reaction conditions used otherwise stated: 2 mM of substrate, 0.1 g/mL of microbial cells, 5 mL of phosphate buffer (100 mM, pH 7.4), and 600 rpm; (**a**) NADH and NADPH were added with the final concentration of 5 mM; (**b**) buffer used: pH 5.0–8.0 phosphate buffer, pH 8.0–9.0 Tris-HCl buffer, pH 9.0–10.0 Gly-NaOH buffer.

Cofactor preference is an important aspect of the biocatalytic reduction of ketones. Many methods have been developed for the regeneration of the coenzyme during the reaction [19,20]. Thus, the cofactor preference of TQ-2 cells was investigated first. As shown in Figure 5a, adding NADPH or NADH into the reaction mixture did not lead to a significant increase in conversion compared with the control. Nevertheless, a slight increase was observed in the presence of NADH. It seems that TQ-2 cells do not have obvious cofactor preference in the biocatalytic conversion of acetophenone. The reason is unknown, as the gene of the related enzyme with alcohol dehydrogenase activity has not been identified and heterologously expressed.

Moreover, the effect of pH on the asymmetric reduction of acetophenone catalyzed by TQ-2 whole cells has also been studied. It was found that TQ-2 cells had a broad pH activity profile and showed good catalytic performance in the pH range of 5.0 to 9.0 (Figure 5b). Interestingly, a conversion of 43% was achieved in Tris-HCl buffer of pH 9.0, implying the high tolerance of TQ-2 cells to high pH conditions. Compared with isolated enzymes, whole-cell biocatalysts often have higher tolerances for harsh reaction conditions. The best conversion of 66% was obtained in 100 mM of phosphate buffer with pH 7.0 for 24 h. Therefore, the optimal pH value of 7.0 was used in the subsequent experiments.

### 2.5. The Effect of Co-Substrate on Microbial Reduction of Acetophenone by B. cereus TQ-2

**Figure 6:**
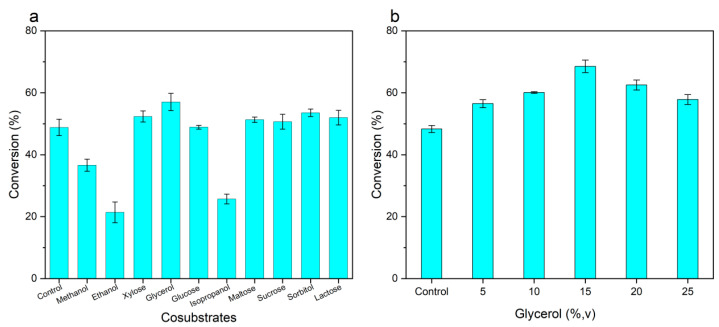
The effect of cosubstrates on the TQ-2 whole cell catalyzed acetophenone reduction. The general reaction conditions used otherwise stated: 2 mM of substrate, 0.1 g/mL of microbial cells, 5 mL of phosphate buffer (100 mM, pH 7.0), and 600 rpm for 48 h; (**a**) 10 mM of each solid cosubstrates or 5% (*v/v*) of each liquid cosubstrates were added; (**b**) glycerol was added with a different final concentration (1–25%, *v/v*). Error bars indicate standard deviation.

The addition of co-substrate for in situ cofactor regeneration is indispensable in the whole cell catalyzed ketone reduction process. Using whole cells as biocatalysts coupled with a co-substrate, such as glucose or glycerol, in ketone asymmetric reduction reactions for chiral alcohols synthesis affords some advantages, especially regarding in situ cofactor regeneration. It has been reported that biocatalytic reactions could proceed smoothly using whole cells in the presence of a co-substrate for coenzyme recycling [22]. In previous studies, different co-substrates have been employed for the regeneration of coenzyme in the bioreduction reactions with various whole-cell biocatalysts. In this study, the effect of several commonly used substances on the bioreduction of acetophenone was examined in order to find a suitable co-substrate for the *B. cereus* TQ-2 cell catalyzed reduction reactions. As illustrated in Figure 6a, the best conversion of 57% was achieved when glycerol was used as a co-substrate. In addition, it was observed that conversion decreased dramatically when methanol, ethanol or isopropanol was used as co-substrates, indicating the severe inhibitory effect of these substances on the catalytic activity of *B. cereus* TQ-2 cells. Presumably, the catalytic activity of related reductase in cells was inhibited by these alcohols. Nevertheless, the detailed reasons need to be further explored by using purified enzymes in the future.

Furthermore, the optimal glycerol concentration for an acetophenone reduction catalyzed by *B. cereus* TQ-2 cells was determined (Figure 6b). It was found that the best conversion of 70% was achieved when the glycerol concentration reached 15% (*v/v*), and further increases did not improve the conversion. The conversion decreased to 54% in the presence of 25% (*v/v*) glycerol. A higher proportion of glycerol may lead to higher viscosity, which could impact the mass transfer of the reaction mixture. Therefore, 15% (*v/v*) of glycerol was selected in the subsequent experiments.

### 2.6. The Effect of Metal Ions on Selective Reduction by TQ-2 Whole Cell Biocatalyst

**Figure 7:**
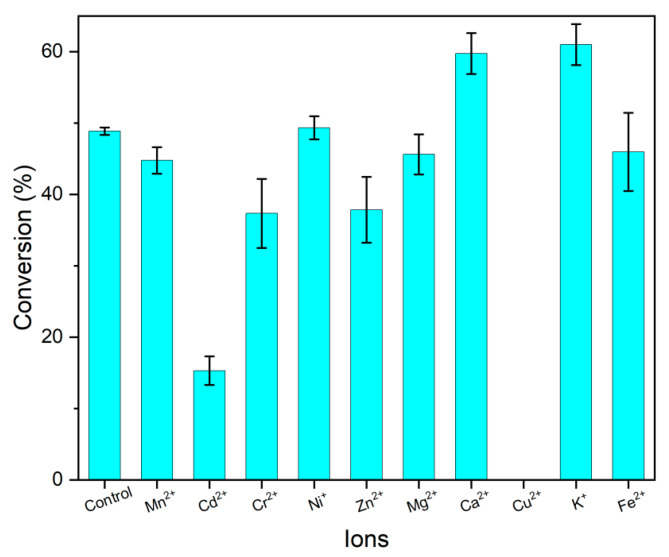
The effect of metal ions on the reduction of acetophenone using catalyzed by *B. cereus* TQ-2 whole cells. The general reaction conditions used otherwise stated: 2 mM of substrate, 0.1 g/mL of microbial cells, 1.0 mM of K^+^, 15% (*v/v*) of glycerol, 5 mL of Tris-HCl buffer (100 mM, pH 8.0), and 600 rpm for 48 h; 1.0 mM of various ions was added. Error bars indicate standard deviation.

It is well established that metal ions play an important role in maintaining the intact structure of some enzymes, which is a prerequisite for their catalytic activities [49]. Thus, the effect of metal ions on the catalytic activity of the *B. cereus* TQ-2 whole-cell biocatalyst was examined. The metal ions were added to the reaction mixture with a final concentration of 1.0 mM. As shown in Figure 7, compared with the control, supplementing the reaction system with 1.0 mM of Ca^2+^ or K^+^ ions significantly enhanced the conversion from 48.9% to 59.7% and 61%, respectively. However, the addition of 1.0 mM of Cd^2+^ dramatically decreased the conversion to 15.3%, which is in agreement with previous reports that demonstrated the negative effect of heavy metal ions on enzyme activity. Moreover, the catalytic activity of TQ-2 whole cells was completely inhibited by the addition of 1.0 mM of Cu^2+^. Nevertheless, another heavy metal ion, Ni^+^, did not have a significant influence on the conversion. A slight decrease in conversion was observed in the presence of 1.0 mM of Mn^2+^, Mg^2+^ or Fe^2+^ ions. It has been reported that many of the alcohol dehydrogenase catalyzing selective reductions of prochiral ketones to enantiopure alcohols are zinc-containing enzymes. However, in this experiment, adding Zn^2+^ ions into the reaction mixture did not improve the conversion, implying that the possible reductase in the TQ-2 cells may not be zinc-dependent.

### 2.7. Bioreduction of Structurally Diverse Carbonyl Compounds by B. cereus TQ-2

Under the optimized reaction conditions, a (*R*)-1-phenylethanol from *B. cereus* TQ-2 whole cell catalyzed selective reduction of acetophenone was successfully obtained, providing a yield of 52% (isolated yield). In addition, in order to expand the applications of the newly discovered *B. cereus* TQ-2 for biocatalytic reductions, the biocatalytic potential of the cells for the synthesis of various alcohols in selective carbonyl compound reductions was explored. As known, alcohols, especially chiral alcohols, are often used as important building blocks in organic synthesis and pharmaceutical applications. In this study, a set of structurally different ketones and aldehydes was used in reduction reactions. As shown in Table 2, the *B. cereus* TQ-2 cells exhibited considerable activity towards the tested compounds with different structures, leading to corresponding alcohol products without byproducts. Compounds **1a**–**1k** were converted to corresponding chiral alcohol products with a 30% to 100% conversion, yielding modest to excellent stereoselectivity.

The activity and stereoselectivity of *B. cereus* TQ-2 were significantly influenced by substituents on the benzene ring of acetophenone. For example, substitution with the electron-withdrawing substituents on the para-position of the ketone resulted in a slight decrease in conversion (**1b**, **1c** and **1d**) and *ee* values (**1c**, **1d**). Interestingly, excellent selectivity and conversion were achieved for compounds **1h** and **1i**, yielding valuable enantiomerically pure *R*-selective alcohols with 99% *ee*. However, the substitution on the ortho-position or meta-position of acetophenone resulted in no conversions during the asymmetric reductions (**1q**, **1r**, **1s** and **1t**). It seemed that the substituents at the ortho-position and/or meta-position severely prevented the occurrence of a reaction. A similar phenomenon was reported for a newly isolated ChKRED20 from *Chryseobacterium* sp. CA49 [7]. The isolation and characterization of the reductases from *B.cereus* TQ-2 cells would provide a good explanation of the catalytic reactions at the molecular level. Moreover, the strain exhibited excellent chemoselective activity towards structurally different aliphatic and aromatic aldehydes in reduction reactions, achieving a >95% conversion, except for **1l**. The bioreduction of aldehyde by *B. cereus* TQ-2 provides a useful alternative to chemical methods for the production of primary alcohols which can be further oxidized to their respective carboxylic acids.

## 3. Materials and Methods

### 3.1. Chemicals and Microorganisms

Acetophenone, acetophenone derivatives and the other chemical reagents involved in this study were purchased from commercial suppliers and used directly without further purification. (*R*)-(−)-α-(Trifluoromethyl) benzyl alcohol, (*R*)-(+)-1-Phenyl-1-butanol, and (*R*)-(−)-4-Phenyl-2-butanol were purchased from Sigma Aldrich Trading Co., Ltd. (Shanghai, China). NADH and NADPH disodium salt were purchased from Aladdin Bio-Chem Technology Co., Ltd. (Shanghai, China) and Bide Pharmatech Ltd. (Shanghai, China), respectively. LB medium and the other media for bacteria enrichment and cultivation were prepared following standard protocols.

### 3.2. Screening and Cultivation of Bacillus cereus TQ-2 Strain

Soil samples collected from the forest of Wutong Mountain located in Shenzhen City in the Guangdong Province of China were used to screen for microorganisms with reduction activity to the model substrate of acetophenone. The bacteria were isolated following a conventional enrichment cultivation procedure. Mineral salt medium (MSM) supplemented with 1 g/L of acetophenone was used for the subsequent enrichment experiments. The entire enrichment process followed the protocol reported by Li et al., with slight modification [34]. Namely, 5 g of soil sample was suspended in 50 mL of deionized water, and 3 mL of suspension was added to 100 mL of the MSM medium containing 1 g/L of acetophenone. The inoculated medium was cultivated at 30 °C, 200 rpm for 3 days. Then, 3 mL of the cultures was added into the fresh MSM medium supplemented with 1 g/L of acetophenone. After repeating the procedure 5 times, the resultant cultures with gradient dilution were spread onto MSM agar plates supplemented with acetophenone. The single colony on the agar plate was picked into 10 mL MSM medium containing 1 g/L acetophenone. After cultivation at 30 °C and 200 rpm overnight, the cultures were stored at −80 °C after being supplemented with 30% (*v/v*) of glycerol.

The isolated bacteria were grown at 30 °C, 200 rpm for 20 h in LB medium composed of tryptone 10.0 g/L, yeast extract 5.0 g/L, NaCl 10.0 g/L. The cells of bacteria were harvested and examined for the reduction activity using acetophenone as substrate following the procedure described in Section 2.4.

### 3.3. Identification of Bacillus cereus TQ-2

The isolated strains with good catalytic performance in transforming acetophenone into the corresponding alcohol were submitted to the China Center Type Culture Collection (CCTCC) for identification based on their physiological and biochemical characters and 16S rDNA sequence. The Bacillus cereus TQ-2 strain was deposited in CCTCC (Wuhan, China) with the accession number CTCC JD 2019287. Phylogenetic trees of the aligned sequences and the closest 16S rDNA matches were constructed using a neighbor-joining method using MEGA 5 with 1000 bootstrap replicates. The 16S rDNA sequence of *B. cereus* TQ-2 was deposited in the Genbank database, with the accession number OP035935.

### 3.4. General Procedure for the Biocatalytic Reduction of Substrates with the Resting Cells of B. cereus TQ-2

The glycerol stock of *B. cereus* TQ-2 was inoculated into 5 mL of LB liquid media for overnight cultivation at 30 °C with shanking at 200 rpm. Then, 1 mL of the overnight-cultivated culture was added to 100 mL of fresh LB liquid media for cultivation under the same growth conditions. After 24 h, cell pellets were collected by centrifugation and washing twice with 100 mM, pH 7.4, phosphate buffer.

The biotransformation of acetophenone and other carbonyl substrates was carried out in a 50-mL screw-capped Falcon centrifuge tube. The reaction mixture was prepared as follows: 5 mL of phosphate buffer (100 mM, pH 7.4) contained 0.5 g of wet cells and 2 mM of acetophenone or other carbonyl substrates with 1% (*v/v*) acetonitrile as co-solvent. After incubation at 30 °C and 600 rpm for 24 h, the transformation was terminated by adding 2 mL of ethyl acetate into the reaction mixture. The organic phase was extracted and filtered for GC analysis.

### 3.5. Analytical Methods

The products of the reactions were measured using the Agilent 8860 GC system instrument with a flame ionization detector. The product identification was performed through comparison with authentic compounds from commercial suppliers and analyzed by GC chromatography. The conversions of substrates were measured with an HP-5MS column (30 m × 0.32 mm × 0.25 μm) using the following programs: Program A (**1l**): Injector 250 °C, Detector 250 °C; 80 °C (1 min) 6 °C/min, 120 °C (1 min). Program B (**1m**, **1n**, **1o**, **1p**): Injector 270 °C, Detector 300 °C; 60 °C (4.5 min), 20 °C/min, 80 °C (1 min) 20 °C/min, 280 °C (5 min).

The separation of enantiomers of chiral alcohols was performed using a chiral Cyclodex-B column (30 m × 0.25 mm × 0.25 μm) using the following programs: Program C (**1a**–**1e**, **1i**): Injector 220 °C, Detector 220 °C; 80 °C (0.5 min), 10 °C/min, 110 °C (5 min), 10 °C/min, 130 °C (6 min), 25 °C/min, 180 °C (0.5 min). Program D (**1g**): Injector 220 °C, Detector 220 °C; 80 °C (1 min), 10 °C/min, 110 °C (5 min), 10 °C/min, 130 °C (6 min), 25 °C/min, 180 °C (5 min), 25 °C/min, 200 °C (1 min). Program E (**1h**, **1j**): Injector 230 °C, Detector 230 °C; 80 °C (1 min), 10 °C/min, 110 °C (5 min), 10 °C/min, 130 °C (6 min), 10 °C/min, 180 °C (5 min), 25 °C/min, 200 °C (4 min).

The transformation of **1f** was analyzed using CP-Chirasil-Dex CB (25 m × 0.25 mm × 0.25 μm). Injector 250 °C, Detector 250 °C; 90 °C (5 min), 3 °C/min, 105 °C, 5 °C/min, 120 °C, 20 °C/min, 180 °C.

The analysis of **1k** transformation was performed on a Chiralcel OD-H column (Daicel Chem. Ind. Ltd. Minato-ku, Tokyo, Japan, 4.6 × 250 mm) using n-heptane: isopropanol (98:2, *v/v*) as a mobile phase at a flow rate of 1.0 mL/min at 25 °C and monitored with a detector at 254 nm. The absolute configuration of the alcohol products was determined through the comparison of their retention time with the related literature or authentic compounds obtained from commercial sources. All of the experiments were performed in triplicate and the mean values are presented.

## 4. Conclusions

Herein, Bacillus cereus TQ-2, a new whole-cell biocatalyst with excellent anti-Prelog stereoselectivity for the asymmetric reduction of prochiral ketones was successfully isolated from a soil sample. The resting cells of the strain showed considerable activity over a broad temperature range and remarkable pH adaptability. Moreover, it was found that the cells exhibit a broad substrate spectrum and can utilize glycerol as a co-substrate for coenzyme regeneration. Thus, many applications of *B. cereus* TQ-2 cells are now possible in future studies. This is the first report of a strain from *B. cereus* species with anti-Prelog stereoselectivity for use in prochiral ketone reduction. Our study provides a whole-cell biocatalyst that can be used in asymmetric ketone reduction, setting the stage for future work concerning the isolation and identification of related enzymes.

## Figures and Tables

**Table 1:** Identification and phenotypic characteristics of TQ-2 strain.

Characteristics	TQ-2	Characteristics	TQ-2
*β*-galactosidase	-	H_2_S production	-
Arginine dihydrolase	+	Urease	-
Lysine decarboxylase	-	Tryptophan deaminase	-
Ornithine decarboxylase	-	Indole production	-
Citric acid utilization	W	3-hydroxy butanone	+
Gelatinase	+	Esculin	+
Glycerine	-	Salicyl alcohol	+
Erythritol	-	Cellose	-
d-arabinose	-	Maltose	+
l-arabinose	-	Lactose	-
Ribose	+	Melibiose	-
d-xylose	-	Saccharose	-
l-xylose	-	Trehalose	+
Adonitol	-	Inulin	-
β-methyl-d-xylitoside	-	Melezitose	-
Galactose	-	Raffinose	-
Glucose	+	Starch	-
Fructose	+	Glycogen	-
Mannose	-	xylitol	-
Sorbose	-	Geraniol	-
Rhamnose	-	d-turanose	-
Dulcitol	-	d-lyxose	-
Inositol	-	d-tagatose	-
Mannitol	-	d-fucose	-
Sorbitol	-	l-fucose	-
Methyl α-d-mannopyranoside	-	d-arabinitol	-
Methyl α-d-Glucopyranoside	-	l-arabinitol	-
*N*-acetylglucosamine	+	Gluconate	-
Amygdalin	-	2-keto-gluconate	-
Arbutin	+	5-keto-gluconate	-

+: Positive; -: Negative; W: weakly positive.

**Table 2:** Reduction of various ketones or aldehydes by *B. cereus* TQ-2 whole cell biocatalyst.

Entry	Substrates	Products	Conversion (%)	*ee* (%)	Selectivity
**1a**	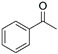	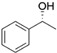	70	99	*R*
**1b**	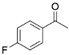	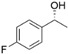	55	99	*R*
**1c**	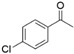	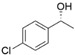	65	75	*R*
**1d**	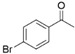	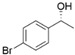	60	66	*R*
**1e**	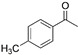	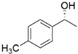	35	99	*R*
**1f**	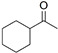	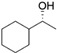	60	60	*R*
**1g**	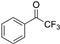	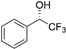	100	40	*S*
**1h**	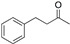	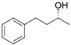	87	99	*R*
**1i**	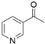	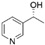	90	99	*R*
**1j**	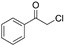	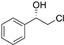	30	80	*S*
**1k**	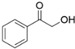	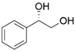	80	50	*S*
**1l**	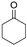	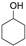	66	-	-
**1m**	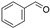	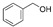	100	-	-
**1n**	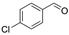	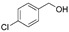	98	-	-
**1o**	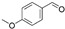	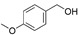	100	-	-
**1p**	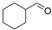	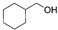	100	-	-
**1q**	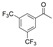	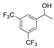	Nr ^a^	-	-
**1r**	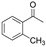	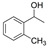	Nr ^a^	-	-
**1s**	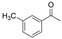	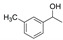	Nr ^a^	-	-
**1t**	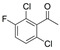	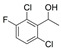	Nr ^a^	-	-

Reaction conditions: 2 mM of substrate, 0.1 g/mL of microbial cells, 15% (*v/v*) of glycerol, 1.0 mM of K^+^, 5 mL of phosphate buffer (100 mM, pH 7.0), and 600 rpm for 24 h. ^a^: No reaction was observed.

## Data Availability

Not applicable.

## Supplementary Materials

The following supporting information can be downloaded at: https://www.mdpi.com/article/10.3390/molecules28031422/s1, Figure S1: 16S rDNA sequence of *B. cereus* TQ-2; Figure S2: The gene sequence of gyrB gene in *B. cereus* TQ-2; Figure S3: MALDI-TOF mass spectrum of *B. cereus* TQ-2 proteins for microbial identification; Figure S4: Consumption of substrate with longer reaction time; Table S1: The blast result of *B. cereus* TQ-2 16S rDNA sequence; Table S2: The blast result of gyrB gene from *B. cereus* TQ-2.
