# Supplementary material for: New Anti-Prelog Stereospecific Whole-Cell Biocatalyst for Asymmetric Reduction of Prochiral Ketones
Source: Molecules. 2023 Feb 2;28(3):1422. doi: 10.3390/molecules28031422 (PMC9921870; doi:10.3390/molecules28031422)
Supplement: Supplementary file 1 [file molecules-28-01422-s001.zip › molecules-2171715-supplementary.pdf]

Supplementary Information

New Anti-Prelog Stereospecific Whole-Cell Biocatalyst for Asymmetric Reduction of Prochiral Ketones

*Min-Yu Wang 1,†, Shun-Ju Cai 1,†, Jia-Chun Lin 1, Xiao-Jun Ji 2 and Zhi-Gang Zhang 1,**

1 School of Pharmaceutical Sciences, Nanjing Tech University, Nanjing 211800, China

2 College of Biotechnology and Pharmaceutical Engineering, Nanjing Tech University, Nanjing 211800, China

** Correspondence: zhangzg@njtech.edu.cn*

† These authors contributed equally to this work.

Figure S1. 16S rDNA sequence of *B. cereus* TQ-2

Figure S2. The gene sequence of *gyrB* gene in *B. cereus* TQ-2

Figure S3. MALDI-TOF mass spectrum of *B. cereus* TQ-2 proteins for microbial identification

Figure S4. Consumption of substrate in reaction with longer reaction time.

Table S1. The blast result of *B. cereus* TQ-2 16S rDNA sequence

Table S2. The Blast result of *gyrB* gene from *B. cereus* TQ-2

TGCAAGTCGAGCGAATGGATTAAGAGCTTGCTCTTATGAAGTTAGCGGCGGACGGGTGAGTAACACG
TGGGTAACCTGCCCATAAGACTGGGATAACTCCGGGAAACCGGGGCTAATACCGGATAACATTTTGA
ACCGCATGGTTCGAAATTGAAAGGCGGCTTCGGCTGTCACTTATGGATGGACCCGCGTCGCATTAGC
TAGTTGGTGAGGTAACGGCTCACCAAGGCAACGATGCGTAGCCGACCTGAGAGGGTGATCGGCCACA
CTGGGACTGAGACACGGCCCAGACTCCTACGGGAGGCAGCAGTAGGGAATCTTCCGCAATGGACGAA
AGTCTGACGGAGCAACGCCGCGTGAGTGATGAAGGCTTTCGGGTCGTAAAACTCTGTTGTTAGGGAA
GAACAAGTGCTAGTTGAATAAGCTGGCACCTTGACGGTACCTAACCAGAAAGCCACGGCTAACTACG
TGCCAGCAGCCGCGGTAATACGTAGGTGGCAAGCGTTATCCGGAATTATTGGGCGTAAAGCGCGCGC
AGGTGGTTTCTTAAGTCTGATGTGAAAGCCCACGGCTCAACCGTGGAGGGTCATTGGAAACTGGGAG
ACTTGAGTGCAGAAGAGGAAAGTGGAATTCCATGTGTAGCGGTGAAATGCGTAGAGATATGGAGGA
ACACCAGTGGCGAAGGCGACTTTCTGGTCTGTAACTGACACTGAGGCGCGAAAGCGTGGGGAGCAAA
CAGGATTAGATACCCTGGTAGTCCACGCCGTAAACGATGAGTGCTAAGTGTTAGAGGGTTTCCGCCC
TTTAGTGCTGAAGTTAACGCATTAAGCACTCCGCCTGGGGAGTACGGCCGCAAGGCTGAAACTCAAA
GGAATTGACGGGGGCCCGCACAAGCGGTGGAGCATGTGGTTTAATTCGAAGCAACGCGAAGAACCTT
ACCAGGTCTTGACATCCTCTGAAAACCCTAGAGATAGGGCTTCTCCTTCGGGAGCAGAGTGACAGGT
GGTGCATGGTTGTCGTCAGCTCGTGTCGTGAGATGTTGGGTTAAGTCCCGCAACGAGCGCAACCCTT
GATCTTAGTTGCCATCATTAAGTTGGGCACTCTAAGGTGACTGCCGGTGACAAACCGGAGGAAGGTG
GGGATGACGTCAAATCATCATGCCCCTTATGACCTGGGCTACACACGTGCTACAATGGACGGTACAA
AGAGCTGCAAGACCGCGAGGTGGAGCTAATCTCATAAAACCGTTCTCAGTTCGGATTGTAGGCTGCA
ACTCGCCTACATGAAGCTGGAATCGCTAGTAATCGCGGATCAGCATGCCGCGGTGAATACGTTCCCG
GGCCTTGTACACACCGCCCGTCACACCACGAGAGTTTGTAACACCCGAAGTCGGTGGGGTAACCTTT
TGGAGCCAGCCGCCTAAGGTGACA

Figure S1. 16S rDNA sequence of *B. cereus* TQ-2

TAATTGGTGAACCACGATCAAACAGGAACAATAACTCGCTTTAAGCCGGATCCAGAGA
TTTTTAAAGAGACGACAGAATATGAATTTGATACGTTAGCGACTCGTATGCGTGAATTA
GCATTTTTAAATCGTAATATTAAATTAACAATTGAAGATAAGCGTGAACATAAGCAAA
AGAAAGAATTCCATTATGAAGGTGGAATTAAATCATATGTTGAGCTATTTAAATCGCTC
AAAACTAACCGAATTCATGAAGAGCCTGTGTATGTAGAAGGGTCAAAAGATGGTATTC
AAGTTGAGGTTTCCTTGCAGTATAACGAAGGATATACAAATAATATTTACTCATTTACG
AATAACATCATACGTATGAA

Figure S2. The gene sequence of *gyrB* gene in *B. cereus* TQ-2

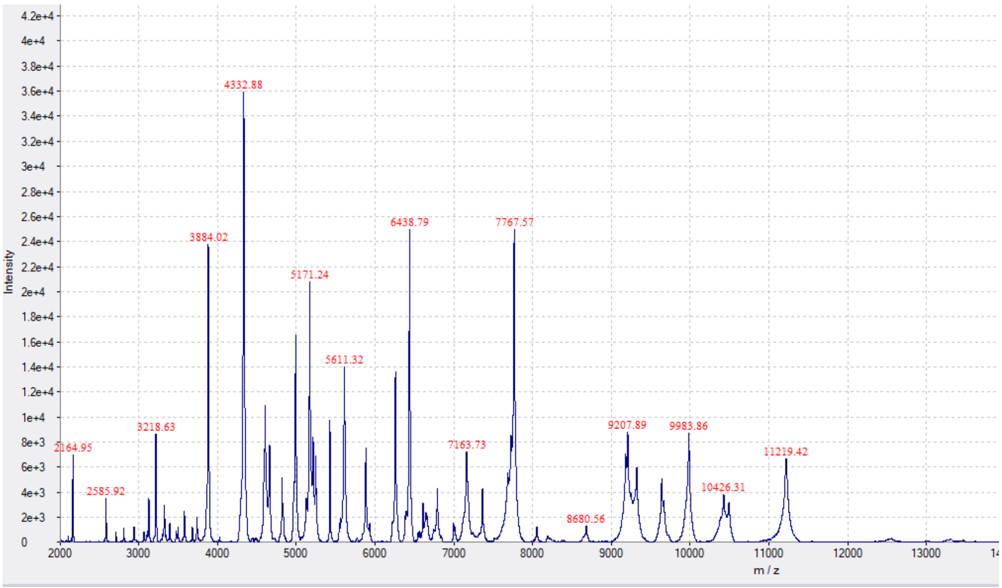

Figure S3. MALDI-TOF mass spectrum of *B. cereus* TQ-2 strain proteins for microbial identification

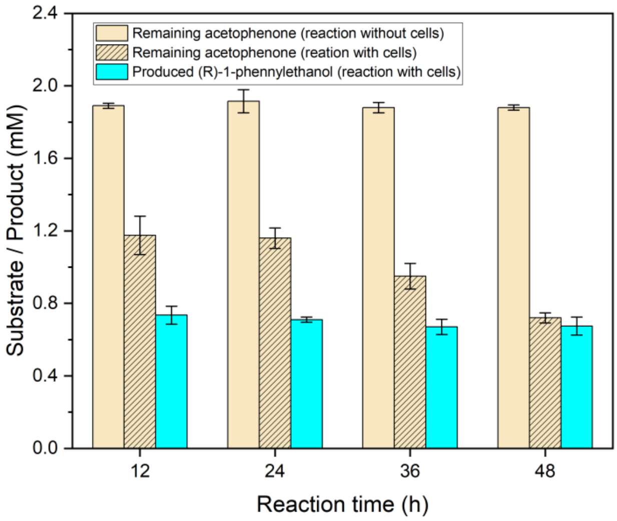

Figure S4. Consumption of substrate in reaction with longer reaction time. Reactions without whole-cell biocatalyst were performed as control for comparison.

Table S1. The blast result of *B. cereus* TQ-2 16S rDNA sequence

Rank	Name	Strain	Accession	Pairwise Similarity (%)	Mismatch/Total nt
1	*Bacillus cereus*	ATCC 14579	AE016877	99.86014	2/1430
2	*Bacillus wiedmannii*	FSL W8-0169	LOBC01000053	99.79021	3/1430
3	*Bacillus paramycoides*	NH24A2	MAOI01000012	99.79021	3/1430
4	*Bacillus paranthracis*	Mn5	MACE01000012	99.79021	3/1430
5	*Bacillus albus*	N35-10-2	MAOE01000087	99.79021	3/1430
6	*Bacillus luti*	TD41	MACI01000041	99.79021	3/1430
7	*Bacillus nitratireducens*	4049	KJ812430	99.79021	3/1430
8	*Bacillus proteolyticus*	TD42	MACH01000033	99.72028	4/1430
9	*Bacillus tropicus*	N24	MACG01000025	99.72028	4/1430
10	*Bacillus anthracis*	Ames	AE016879	99.72028	4/1430
11	*Bacillus pacificus*	EB422	KJ812450	99.65035	5/1430
12	*Bacillus toyonensis*	BCT-7112	CP006863	99.58042	6/1430
13	*Bacillus mobilis*	0711P9-1	MACF01000036	99.58042	6/1430
14	*Bacillus pseudomycoides*	DSM 12442	ACMX01000133	99.44056	8/1430
15	*Bacillus mycoides*	DSM 2048	ACMU01000002	99.37063	9/1430
16	*Bacillus cytotoxicus*	NVH 391-98	CP000764	97.83217	31/1430
17	*Bacillus oryzaecorticis*	R1	KF548480	96.28623	41/1104
18	*Bacillus tianshenii*	YIM M13235	KF811034	95.51507	64/1427
19	*Bacillus marisflavi*	JCM 11544	LGUE01000011	95.44818	65/1428
20	*Bacillus halmapalus*	DSM 8723	KV917375	95.37491	66/1427

Table S2. The Blast result of *gyrB* gene from *B. cereus* TQ-2

Accession	Description	Max Score	Total Score	Query Cover (%)	E value	Per. Ident. (%)
AF136388.1	*Bacillus cereus* strain H2 gyrase B subunit (gyrB) gene, partial cds	529	529	99	4.00E-146	92.72
CP015589.1	*Bacillus cereus* strain AR156, complete genome	523	523	99	2.00E-144	92.45
CP045777.1	*Bacillus paranthracis* strain CFSAN068816 chromosome, complete genome	518	518	99	9.00E-143	92.18
CP026375.1	*Bacillus cereus* ATCC 10987 chromosome	518	518	99	9.00E-143	92.18
CP026376.1	*Bacillus cereus* strain G9241 chromosome	518	518	99	9.00E-143	92.18
CP023726.1	*Bacillus cereus* strain BHU2 chromosome	518	518	99	9.00E-143	92.18
CP023179.1	*Bacillus cereus* strain CC-1 chromosome, complete genome	518	518	99	9.00E-143	92.18
CP016316.1	*Bacillus cereus* strain M3, complete sequence	518	518	99	9.00E-143	92.18
CP020937.1	*Bacillus cereus* strain BC-AK genomic sequence	518	518	99	9.00E-143	92.18
CP009590.1	*Bacillus cereus* G9241, complete genome	518	518	99	9.00E-143	92.18
CP009369.1	*Bacillus cereus* strain FM1, complete genome	518	518	99	9.00E-143	92.18
